# Immunomodulatory Therapeutic Strategies in Stroke

**DOI:** 10.3389/fphar.2019.00630

**Published:** 2019-06-20

**Authors:** Kyle Malone, Sylvie Amu, Anne C. Moore, Christian Waeber

**Affiliations:** ^1^Department of Pharmacology and Therapeutics, School of Pharmacy, University College Cork, Cork, Ireland; ^2^Cancer Research @UCC, University College Cork, Cork, Ireland; ^3^School of Biochemistry and Cell Biology, University College Cork, Cork, Ireland

**Keywords:** stroke, immunomodulation, ischaemia, immune, therapy, neuroinflammation

## Abstract

The role of immunity in all stages of stroke is increasingly being recognized, from the pathogenesis of risk factors to tissue repair, leading to the investigation of a range of immunomodulatory therapies. In the acute phase of stroke, proposed therapies include drugs targeting pro-inflammatory cytokines, matrix metalloproteinases, and leukocyte infiltration, with a key objective to reduce initial brain cell toxicity. Systemically, the early stages of stroke are also characterized by stroke-induced immunosuppression, where downregulation of host defences predisposes patients to infection. Therefore, strategies to modulate innate immunity post-stroke have garnered greater attention. A complementary objective is to reduce longer-term sequelae by focusing on adaptive immunity. Following stroke onset, the integrity of the blood–brain barrier is compromised, exposing central nervous system (CNS) antigens to systemic adaptive immune recognition, potentially inducing autoimmunity. Some pre-clinical efforts have been made to tolerize the immune system to CNS antigens pre-stroke. Separately, immune cell populations that exhibit a regulatory phenotype (T- and B- regulatory cells) have been shown to ameliorate post-stroke inflammation and contribute to tissue repair. Cell-based therapies, established in oncology and transplantation, could become a strategy to treat the acute and chronic stages of stroke. Furthermore, a role for the gut microbiota in ischaemic injury has received attention. Finally, the immune system may play a role in remote ischaemic preconditioning-mediated neuroprotection against stroke. The development of stroke therapies involving organs distant to the infarct site, therefore, should not be overlooked. This review will discuss the immune mechanisms of various therapeutic strategies, surveying published data and discussing more theoretical mechanisms of action that have yet to be exploited.

## Introduction

Stroke remains a major cause of morbidity and mortality worldwide (Krishnamurthi et al., 2013). Despite the clear impact of acute ischaemic stroke (AIS) on patients, recombinant tissue plasminogen activator (tPA) is the only medication specifically approved for its treatment. Beyond a therapeutic window of 4.5 h, however, the benefits of thrombolysis are outweighed by its risks, with the incidence of haemorrhagic transformation increasing dramatically (Lees et al., 2010). Consequently, only a small percentage of patients receive tPA. Separately, mechanical thrombectomy is indicated for up to 6 h after the onset of ischaemic stroke symptoms, though recent trials have allowed for an extension to 24 h in selected patients (Robinson, 2018). Additional agents for the treatment of AIS are now required, not only as adjuncts to increase the therapeutic potential of thrombolysis and thrombectomy, but also for patients who, for various reasons, cannot receive standard care. Ideally, such therapies, through curtailing damage to the brain post-stroke or contributing to long-term tissue remodelling, would improve patient survival and functional outcomes.

In the last few decades, hundreds of agents targeting numerous pathophysiological mechanisms have failed clinical trials (O’Collins et al., 2006). Yet from this research, important lessons have been learned. The role of immunity in all stages of stroke, for instance, is becoming better understood. Consequently, a range of immune-targeted therapies are now in development and testing (Malone et al., 2018).

A number of reviews have examined the individual components of the immune response to stroke, or the immunomodulatory agents currently enrolled in stroke clinical trials (Iadecola and Anrather, 2011; Smith et al., 2015; Drieu et al., 2018). However, many aspects of the immune response play multifaceted roles in stroke, and the interaction between the brain and immune system post-stroke is an ever-evolving picture. Future stroke treatment may involve a range of mechanical, immunological, and pharmacological therapies, all working in concert (Neuhaus et al., 2017). If clinically translated, strategies manipulating the immune response could play a crucial part. In this review, we provide an update on immune-targeted strategies in AIS, from the level of pathology to pre-clinical evidence and clinical trials. The aim of this review, therefore, is to discuss immunomodulatory therapeutic strategies in stroke (listed in **Table 1**), surveying published data and highlighting more theoretical mechanisms of action that have yet to be explored.

**Table 1:** Immunomodulatory therapeutic strategies in stroke.

Target	Role in stroke pathology	Proposed therapy
Astrocytes	—Promote neurotoxicity through pro-inflammatory cytokine release (e.g., IL-15) and glial scar formation (Roy-O’Reilly and McCullough, 2017). —Provide neuroprotection *via* reduced excitotoxicity, neurotrophin production, and angiogenic and synaptogenic effects (Wang et al., 2018).	—CDK5-knockdown astrocyte cell therapy (Becerra-Calixto and Cardona-Gómez, 2017)
Macrophage/microglia	—Increase ischaemic injury (M1 type) *via* release of ROS, NO, and pro-inflammatory cytokines (e.g., TNF-α and IL-12) (Chiba and Umegaki, 2013). —Promote tissue repair (M2 type) *via* growth factors, anti-inflammatory cytokines (e.g., IL-4), and phagocytosis of dead cells (Kanazawa et al., 2017).	—Minocycline (macrophage deactivator) (Lampl et al., 2007) —Edaravone (free radical scavenger) (Chen et al., 2014)
Complement pathway	—Contributes to neuronal cell death through C3a and C5a anaphylatoxins (Alawieh et al., 2015a). —Contributes to subacute/chronic neurogenesis and repair (Alawieh and Tomlinson, 2016).	—CR2-Crry (complement inhibitor) (Alawieh et al., 2015b) —B4Crry (complement inhibitor) (Alawieh et al., 2018)
Nitric oxide	—Provides beneficial reductions in platelet aggregation and leukocyte adhesion as well as improved vascular tone and host defence (Kim et al., 2014). —Directly neurotoxic in high levels (Venkataramana et al., 2015).	—Glyceryl trinitrate (nitric oxide donor) (Chen et al., 2017)
IL-1β	—Promotes neurotoxicity through NF-κB-mediated generation of a pro-inflammatory environment (Boutin et al., 2001).	—Anakinra (recombinant IL-1 receptor antagonist) (Smith et al., 2018)
TNF-α	—Promotes BBB breakdown, leukocyte infiltration, and brain oedema (acute stage) (Amantea et al., 2009). —Contributes to neuronal and microvasculature repair (chronic stage) (Amantea et al., 2009).	—Etanercept (TNF-inhibitor) (Tobinick et al., 2014)
COX/LO pathway	—COX: PGE_2_ increases ischaemic injury (Iadecola and Gorelick, 2005). PGE_1_ contributes to post-stroke neurogenesis and angiogenesis (Ling et al., 2016). —LO: LTC4 promotes BBB dysfunction (Baskaya et al., 1996).	—NS398 (COX-2 inhibitor) (Sugimoto and Iadecola, 2003) —Montelukast (cysteinyl leukotriene receptor-1 antagonist) (Saad et al., 2015)
MMPs	—Increased MMP activity causes neuronal apoptosis, BBB breakdown, leukocyte infiltration, and brain oedema (Rosell and Lo, 2008). —Involved in late-stage neovascularization and neurovascular remodelling (Zhao et al., 2006).	—Minocycline (MMP-9 inhibitor) (Murata et al., 2008)
Chemokines	—Involved in acute ischaemic injury *via* increased leukocyte infiltration, ROS production, and BBB disruption (Chen et al., 2018a). —Possible function in chronic stem cell recruitment to the infarct site (Wang et al., 2002).	—NR58-3.14.3 (pan-chemokine inhibitor) (Mirabelli-Badenier et al., 2011)
Adhesion molecules	—Upregulation of selectins, immunoglobulins, and integrins post-stroke increase leukocyte infiltration with resulting infarct growth (Yilmaz and Granger, 2008).	—Enlimomab (anti-ICAM-1 antibody) (Investigators, 2001) —Hu23F2G (anti-Mac-1 antibody) (Becker, 2002) —Natalizumab (anti-VLA-4 antibody) (Elkins et al., 2017) —Fingolimod (S1P receptor modulator) (Zhu et al., 2015)
Regulatory immune cells	—Regulatory B and T-cells provide neuroprotection and enhance post-stroke repair through dampening excessive immune responses and producing anti-inflammatory cytokines (e.g., IL-10) (Liesz and Kleinschnitz, 2016; Bodhankar et al., 2013)	—IL-2/IL-2 antibody complex (Zhang et al., 2018) —Adoptive cell transfer (Ren et al., 2011)

IL-15, interleukin-15; CDK5, cyclin-dependent kinase 5; ROS, reactive oxygen species; NO, nitric oxide; TNF-*α*, tumour necrosis factor alpha; IL-12, interleukin-12; IL-4, interleukin 4; NF-*κ*B, COX, cyclooxygenase; LO, lipoxygenase; BBB, blood brain barrier; PGE2, prostaglandin E2; LTC4, leukotriene C4; MMP, matrix metalloproteinase; S1P, sphingosine 1-phosphate; VLA-4, very late antigen-4; ICAM-1, intercellular adhesion molecule-1.

## Stroke and the Blood–Brain Barrier

The brain was long thought to be hidden from our immune system. This is in large part due to the lack of an obvious lymphatic system and the presence of a blood–brain barrier (BBB). This barrier separates the blood compartment from the central nervous system (CNS) at the level of brain endothelial cells, thus rigorously controlling immune cell trafficking between the CNS and the periphery (Wilson et al., 2010). Correspondingly, breakdown of this barrier may underlie the pathophysiology of immune-based CNS disorders. However, while the BBB is indeed compromised in some CNS disorders and brain injury, the recent discovery of meningeal lymphatics suggests that, even under basal conditions, the “immune privilege” of the brain is not as absolute as was once thought (Louveau et al., 2015).

**Figure 1:**
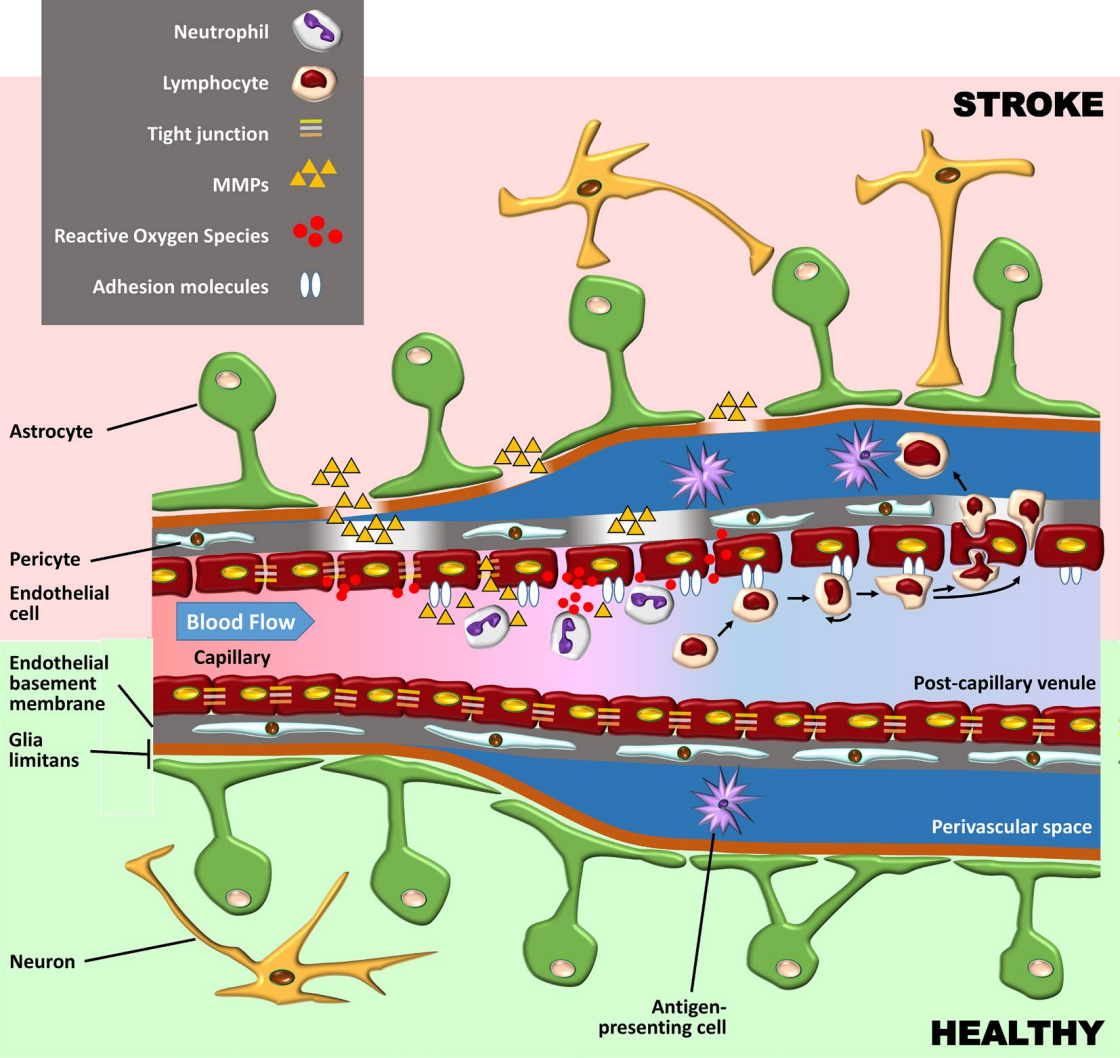
Schematic representation of the blood–brain barrier (BBB) under healthy conditions (bottom part of the figure) and during its early breakdown following stroke (top part). The BBB is composed of a layer of endothelial cells with tight junctions composed of transmembrane and cytoplasmic adhesion molecules. These cells produce a basement membrane containing embedded pericytes. Astrocytes and their end feet constitute the glia limitans perivascularis. At the capillary level (left side of the figure), the endothelial basement membrane and the glia limitans are indistinguishable, but at the level of post-capillary venules (on the right), they are separated by a perivascular space containing cerebrospinal fluid (CSF) and antigen-presenting bone marrow-derived macrophages and dendritic cells. Following reperfusion, reactive oxygen species (ROS) cause an initial breakdown of the BBB by activating matrix metalloproteinases (MMPs) and upregulating inflammatory mediators. Stroke leads to an increased expression of integrin molecules on the leukocyte surface (not shown) and of the corresponding adhesion molecules on the endothelium. Leukocytes tether and roll onto the vascular endothelium before being activated by chemokines (not shown). They then firmly adhere to the endothelium and undergo either transcellular or paracellular diapedesis through the endothelial layer.

The BBB is composed of specialized endothelial cells in capillaries and post-capillary venules of the brain and spinal cord, characterized by low pinocytotic activity and by complex tight junctions composed of transmembrane and cytoplasmic adhesion molecules (Serlin et al., 2015). These endothelial cells produce a basement membrane containing embedded pericytes. Another basement membrane, produced by astrocytes, constitutes together with the astrocytic end feet, the glia limitans perivascularis. While these two basement membranes are indistinguishable at the capillary level, they are separate at the level of post-capillary venules, where they form a perivascular space containing cerebrospinal fluid (CSF) and antigen-presenting cells. Under basal conditions, leukocytes rarely penetrate the glia limitans but accumulate in this perivascular space during inflammation (as well as in the leptomeningeal and ventricular spaces) (**Figure 1**). Here, they fulfil an immune-surveillance function by interacting with antigen-presenting bone marrow-derived macrophages and dendritic cells.

The fact that oxidative stress leads to BBB breakdown has been observed in various neurological diseases, including multiple sclerosis (MS) and stroke (Rosenberg, 2012). Oxidative stress is not associated with the ischaemic episode itself but rather with reperfusion, which, although essential to limit brain injury, also causes an initial breakdown of the BBB by activating matrix metalloproteinases (MMPs) and upregulating inflammatory mediators. In experimental studies, BBB opening is biphasic. The initial BBB breakdown occurs within 2–3 h of stroke onset, is associated with activation of MMP-2, and is accompanied by the development of vasogenic oedema (i.e., excess accumulation of fluid in the brain extracellular spaces). Following a partial recovery of BBB function, a second increase in BBB permeability, occurring 24 to 48 h after stroke, is characterized by inflammatory processes with upregulation of inducible MMPs (MMP-3 and MMP-9) and cyclooxygenase (COX)-2, tight junction redistribution, and neutrophil infiltration (Jiang et al., 2018). It is worth mentioning that the biphasic profile of BBB opening has been questioned (as discussed in Merali et al., 2017), and the BBB might in fact be continuously opened for up to a week.

Although neutrophils are the first subset of leukocytes to appear in the ischaemic brain (detected within the first hour), these neutrophils are not found in parenchyma but remain in the cerebral microvessels, which they occlude (contributing to the no-reflow phenomenon) and from where they can damage the BBB by releasing proteolytic enzymes and reactive oxygen species (ROS). Neutrophils penetrate the CNS parenchyma mainly following the more damaging second opening of the BBB, which leads to severe endothelial damage, gross destruction of adjacent blood vessels, and in some cases haemorrhagic transformation (Perez-de-Puig et al., 2015).

Despite the relatively early opening of the BBB, lymphocytes are only detected within the ischaemic territory 24 h after stroke onset, their number increasing over the following days (Schroeter et al., 1994; Brait et al., 2010). Larger infarctions may be associated with earlier appearance of lymphocytes in the brain (Chu et al., 2014). Following stroke, antigen presentation to lymphocytes can rapidly occur in the spleen and lymph nodes, since BBB opening and cell damage following stroke enable CNS antigens to access the blood compartment early after the onset of ischaemia. Indeed, CNS antigens are found in the cervical lymph nodes 24 h after middle cerebral artery occlusion in mice (van Zwam et al., 2009), and the concentration of these antigens seems to correlate with stroke severity (Jauch et al., 2006). Brain-derived antigens are also found in cervical lymph node cells expressing costimulatory major histocompatibility complex II (MHC-II) receptors in patients with acute stroke (Planas et al., 2012). This suggests that improving BBB function following stroke or at least preventing lymphocytes from entering the brain following their activation with brain antigens might improve functional outcome after stroke. The latter mechanism of action might explain why fingolimod was found to be effective in rodent models of stroke (Wei et al., 2011), reduced the risk of haemorrhagic transformation associated with delayed administration of tPA (Campos et al., 2013), and showed promising results in small clinical trials (Fu et al., 2014a; Fu et al., 2014b).

## Acute Post-Stroke Immune Responses

**Figure 2:**
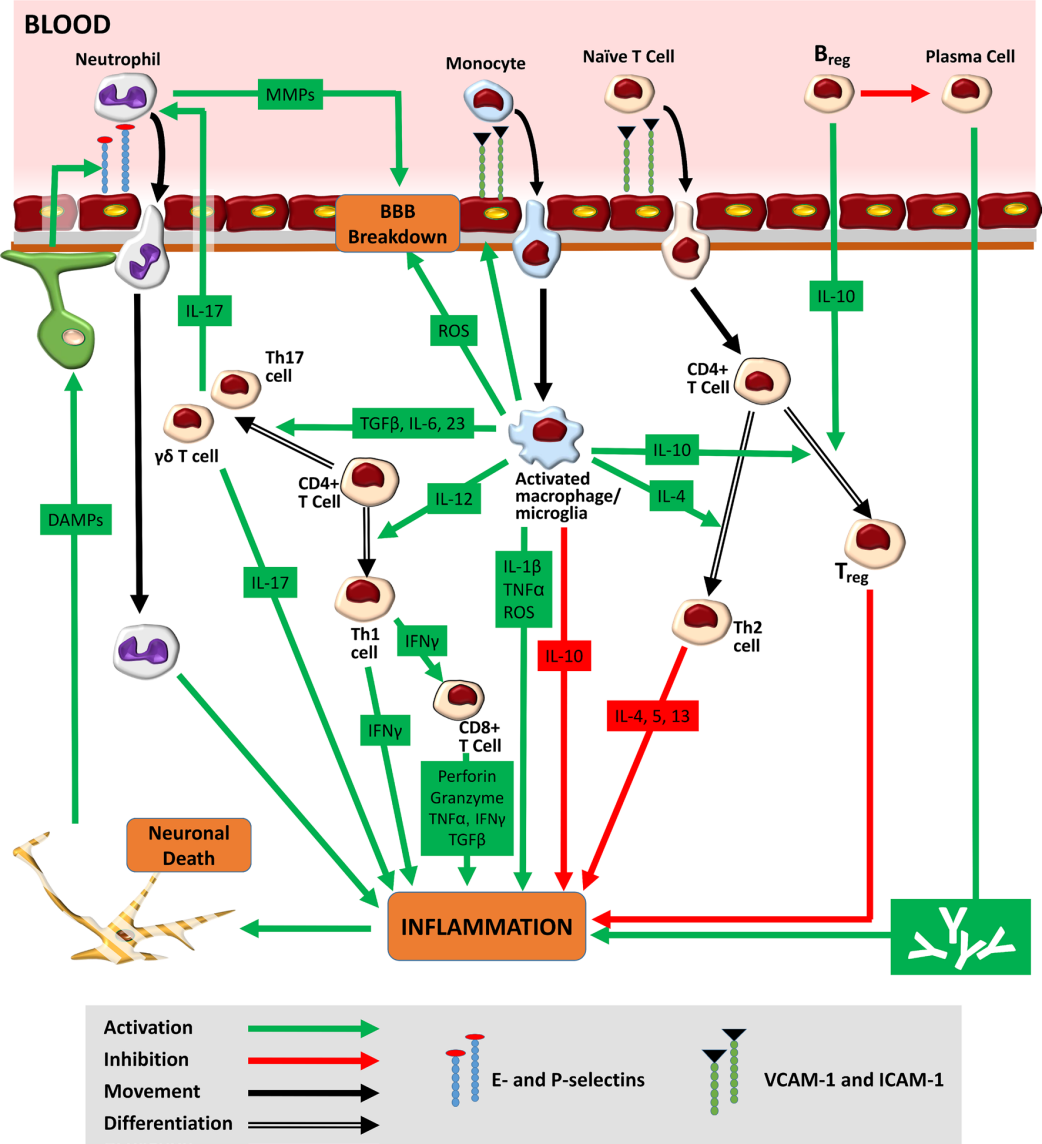
Immune response to stroke. Hypoperfusion causes an immediate deprivation of glucose and oxygen to the brain, leading to widespread neuronal cell death. The release of danger/damage-associated molecular patterns (DAMPs) from dying neurons results in the secondary activation of astrocytes and microglia. The release of chemokines/cytokines from glial cells generates an inflammatory environment featuring ROS, activated leukocytes, and the upregulated expression of adhesion molecules on endothelial cell membranes. Adhesion molecules such as E- and P-selectin mediate the initial tethering of circulating leukocytes to the endothelium. Separate surface molecules such as ICAM-1 and VCAM-1 then facilitate firm adhesion and transmigration. Neutrophils, entering the brain as early as 1 h post-stroke, increase BBB permeability *via* MMPs, further exacerbating ischaemic injury. Monocytes, infiltrating 1–2 days later, function as tissue macrophages. The M1 macrophage/microglia phenotype increases ischaemic injury through the production of ROS and pro-inflammatory cytokines (TNF-α and IL-1β). The M1 subtype also secretes cytokines [IL-12, IL-6, transforming growth factor beta 1 (TGF-β), and IL-23], which encourage the differentiation of infiltrated naïve CD4+ T-cells into pro-inflammatory Th1 and Th17 forms. Th1 cells, through release of interferon gamma (IFNγ), promote the cytotoxic activity of CD8+ T-cells. Th17 cells (as well as their γδ T-cell counterparts) further increase neutrophilic activity and enhance ischaemic through the production of IL-17. Ultimately, the pro-inflammatory milieu seen in the acute stages of ischaemic stroke gives way to a second, subacute anti-inflammatory phase typified by increased M2 microglial/macrophagic activity. The release of IL-10 from both glial cells and circulating Bregs encourages the generation of Tregs, a cell type that promotes neuroprotection and repair. Bregs may also play a role in the chronic immune response to stroke where they serve to reduce the effect of long-term antibody-mediated neurotoxicity.

In 87% of cases, stroke is due to a drastic reduction in cerebral blood flow (CBF) that results in metabolic and functional deficits. At a pathophysiological level, however, a much more complex web of interactions is seen. Following the onset of cerebral ischaemia, an orderly sequence of events involving the CNS, the lymphoid organs, the blood, and the wider vasculature is triggered (Iadecola and Anrather, 2011). Hypoperfusion causes an immediate deprivation of glucose and oxygen to the brain, leading to a fall in ATP production. Within minutes of the ischaemic insult, the downstream processes of acidotoxicity, excitotoxicity, oxidative stress, and inflammation begin, giving rise to widespread neuronal cell death (Fann et al., 2013). The release of danger/damage-associated molecular patterns (DAMPs) from dying neurons induces a new phase of innate inflammatory response (**Figure 2**). Pattern-recognition receptor (PRR) activation on microglia results in the inflammasome-mediated production of interleukin 1-beta (IL-1β) and tumour necrosis factor-α (TNF-α) (Marsh et al., 2009). The subsequent release of cytokines/chemokines from astrocytes and endothelial cells creates an inflammatory environment, containing IL-17, granzyme, ROS, and perforin (Fann et al., 2013). In the vasculature, platelets are activated alongside endothelial cells and proteins of the complement system (Iadecola and Anrather, 2011). Fibrin, the end-product of the coagulation cascade, traps leukocytes and platelets, forming microvascular occlusions (del Zoppo et al., 1991). A fall in the bioavailability of nitric oxide (NO), which normally inhibits platelet aggregation, exacerbates this intravascular plugging (Chen et al., 2017). Capillary occlusions are further promoted by pro-inflammatory signalling, the constriction of pericytes, and the translocation of the adhesion molecule P-selectin to cell membranes. As mentioned above, this combination of oxidative stress, inflammatory cytokines, downregulated endothelial junction proteins, and increased MMP activity increases BBB permeability (Engelhardt and Sorokin, 2009). In the perivascular space, proteins released from macrophages (ROS, IL-1β, and TNF-α) and mast cells (histamine and proteases) further degrade BBB structures (Iadecola and Anrather, 2011). Swelling of endothelial cells first causes protein extravasation and interstitial oedema. Detachment of endothelial cells from the basement membrane then results in unimpeded entry of free water and serum into the brain, ultimately leading to haemorrhage (Petrovic-Djergovic et al., 2016). A compromise in BBB integrity also allows leukocyte entry into the infarct site. Endothelial cell-derived prostaglandins and chemoattractant molecules drive the trafficking process (Engblom et al., 2002; Huang et al., 2006). The infiltration of macrophages, neutrophils, and lymphocytes into the brain is mediated by interactions between high-avidity integrin molecules on the leukocyte surface and the corresponding ligands on the endothelium. The expression of such proteins, including vascular cell adhesion molecule-1 (VCAM-1) and intracellular adhesion molecule-1 (ICAM-1), is upregulated post-stroke (Supanc et al., 2011). Activated leukocytes secrete a mix of collagenases, gelatinases, ROS, cytokines, leukotrienes, and platelet-activating factor, promoting vasoconstriction, platelet aggregation, and further neurotoxicity (Kim et al., 2016). As the ischaemic penumbra expands, a combination of fresh excitotoxic responses, oxidative stress, and mitochondrial dysfunction drives the next phase of both the local and systemic immune responses.

## Therapeutic Strategies Targeting Astrocytes and Microglia

Astrocytes undergo numerous changes post-ischaemia, including rapid swelling, increased intracellular calcium signalling, and upregulated expression of glial fibrillary acidic protein (GFAP) (Petrovic-Djergovic et al., 2016). The astroglial response begins in the infarct site as early as 4 h post-stroke, reaching peak activity around day 4 (Kim et al., 2016). Although this “reactive gliosis” contributes to long-term healing, the initial formation of the glial scar is thought to be detrimental. The scar acts as both a physical and chemical barrier to axonal re-growth, preventing reinnervation (Barreto et al., 2011). Several studies have shown that decreased astrogliosis correlates with reduced infarct size (reviewed in Barreto et al., 2011). Separate research has highlighted how astrocytes can play a similarly detrimental role in AIS as traditional leukocytes, increasing interest in immunomodulatory strategies targeting these cells. Astrocytes have been shown to express various pro-inflammatory mediators in the acute phase including cytokines, chemokines, and inducible nitric oxide synthase (iNOS) (Dong and Benveniste, 2001). Astrocyte-derived IL-15, for example, augments cell-mediated immunity post-stroke, promoting ischaemic injury (Roy-O’Reilly and McCullough, 2017). More recent work, however, points to astrocytes as promising therapeutic targets for neuroprotection and neurorestoration (Liu and Chopp, 2016). Fundamentally, the glial scar divides the site of injury from surrounding viable tissue, hindering infarct growth. During the acute phase, astrocytes also limit neuronal cell death by reducing excitotoxicity and releasing neurotrophins (Liu and Chopp, 2016). Finally, astrocytes contribute to the chronic processes of angiogenesis, neurogenesis, and synaptogenesis (Wang et al., 2018). As for many other immune targets in AIS, the manipulation of the astrocytic response may involve a combination of pharmacological [e.g., cyclin-dependent kinase 5 (CDK5) inhibitors] and cell-based therapies (Becerra-Calixto and Cardona-Gómez, 2017).

In the resting state, microglia exhibit a ramified appearance. However, in the event of acute brain injury, they undergo a morphological transformation to an active amoeboid state, making them virtually indistinguishable from circulating macrophages (Kim et al., 2016). Microglia activate within minutes of brain ischaemia, with products detectable as early as 1 h post-stroke (Xu and Jiang, 2014). Peripheral macrophages infiltrate 1–2 days later, reaching peak levels 3–7 days after the onset of ischaemia (Xu and Jiang, 2014). Overall, microglial activity predominates in the early stages of ischaemia, while blood-derived cells contribute more to the subacute and chronic phases of neuroinflammation. The destructive effects of microglia/macrophages in AIS are well documented (Chiba and Umegaki, 2013). Other studies, however, in which the selective ablation of microglial cells leads to worsened stroke outcomes suggest a protective function (Lalancette-Hébert et al., 2007). Indeed, blocking macrophage infiltration abolished long-term behavioural recovery and led to reduced anti-inflammatory signalling in the brain (Wattananit et al., 2016). Importantly, the overall net effect of microglia/macrophages in AIS depends on the M1/M2 polarization status. It has been demonstrated that the anti-inflammatory M2 phenotype gives way to the pro-inflammatory M1 phenotype during disease progression (Xu and Jiang, 2014). The shift between the M1 and M2 subtypes depends on specific cytokine signals from the local inflammatory milieu. Several molecular switches, such as triggering receptor expressed on myeloid cells 2 (TREM2), have been shown to control the functional polarization in the setting of AIS, making them potential immunomodulatory targets (Kawabori et al., 2015). At pre-clinical level, agents that prevent microglial activation, such as minocycline, hyperbaric oxygen, or the free radical scavenger, edaravone, are neuroprotective, possibly as a result of reduced M1 activity (Chen et al., 2014). Minocycline, a semi-synthetic tetracycline compound, progressed to human trials. Two phase II open-label, placebo-controlled trials concluded that the drug was safe and significantly improved clinical outcome (Lampl et al., 2007; Padma Srivastava et al., 2012). In a third study, however, minocycline did not prove efficacious (Kohler et al., 2013). Evidently, a more nuanced understanding of the M1/M2 phenomenon in AIS is needed, alongside a greater appreciation of the differences between resident microglia and circulating macrophages. Some authors contend that microglia/macrophages may yet prove an attractive immunomodulatory target in the context of cell-based therapies (Kanazawa et al., 2017).

## Therapeutic Strategies Targeting Inflammatory Mediators

### The Complement Pathway

The proteins and receptors of the complement pathway, once thought to be restricted to immune cells, are now known to be expressed by neurons, microglia, and astrocytes (Alawieh et al., 2015a). The complement pathway contributes to neuroinflammation in the initial phase of AIS (Iadecola and Anrather, 2011), but other studies indicate a second function for these proteins in the subacute and chronic stages, predominantly in post-stroke neurogenesis and repair (Alawieh and Tomlinson, 2016). As with many facets of the immune response to stroke, the dual role of complement has hindered the development of effective therapies. A number of pre-clinical studies have shown that the anaphylatoxins C3a and C5a promote tissue inflammation and neurotoxicity. Administration of a C3a receptor antagonist in a mouse model of stroke, for example, reduced infarct size and improved neurological outcome, while similar results were seen in C3a^−/−^ mice (Mocco et al., 2006a). C3a receptor antagonism has also been shown to promote neuroblast formation, curb T-cell infiltration, and lead to better overall histologic and functional outcomes (Ducruet et al., 2012). Use of a C5a receptor blocker, meanwhile, significantly reduced infarct volume and improved neurological outcomes 24 h after ischaemia (Kim et al., 2008). Evidence also exists for a pathological role for complement in human stroke. Patients deficient in mannose-binding lectin, for example, display smaller infarct size (Osthoff et al., 2011). The plasma levels of complement proteins are also raised following AIS (Cojocaru et al., 2008). Despite these findings, none of the complement inhibitors tested pre-clinically have progressed to clinical trials. Several concerns remain around these drugs including poor bioavailability, questionable efficacy, increased risk of infection, and potential interference with homeostatic function. As a result, several other complement receptor-based inhibitors [e.g., soluble complement receptor 1 (sCR1) and sialyl Lewis x glycosylated soluble complement receptor 1 (sCR1sLe^x^)] have been developed. However, while sCR1 reduced ischaemic injury in mice, it failed to provide the same neuroprotection in a non-human primate model of stroke (Huang et al., 1999; Mocco et al., 2006b). Similarly, while sCR1sLe^x^ decreased cerebral infarct volume and inhibited platelet/neutrophil accumulation in mice, the effect was not replicated in either rats or non-human primates (Huang et al., 1999; Ducruet et al., 2007). No further development of sCR1sLe^x^ has been reported. More promise has been shown by compounds attached to a recombinant form of complement receptor type 2 (CR2). Both CR2-Crry (an inhibitor of all complement pathways) and CR2-fH (an inhibitor of the alternative pathway) significantly reduced infarct size, apoptotic cell death, and neurological deficits in a mouse model of transient middle cerebral artery occlusion (tMCAO) (Alawieh et al., 2015b). The same research group observed improved long-term motor and cognitive recovery using B4Crry, another site-targeted inhibitor (Alawieh et al., 2018). Separately, recent evidence has shown that tPA upregulates complement cascade activity in ischaemic stroke models through plasmin-mediated cleavage of the C3 protein. The resulting C3a anaphylatoxin promotes post-ischaemic brain haemorrhage and cerebral oedema. Inhibition of the C3a receptor, however, abrogates such adverse effects, indicating a potential role for complement inhibitors as adjuncts to tPA. Currently, the risk of haemorrhagic transformation limits the clinical use of thrombolysis. However, complement inhibition could enhance the therapeutic window of tPA through the mechanism described above (Zhao et al., 2017).

### Nitric Oxide

The protective functions of NO in AIS include regulation of vascular tone, inhibition of platelet aggregation, prevention of leukocyte adhesion, and host defence (Kim et al., 2014). High levels of nitric oxide, however, have been shown to be neurotoxic (Venkataramana et al., 2015). NO is generated *in vivo* through nitric oxide synthase (NOS), and though all three isoforms (endothelial NOS, neuronal NOS, and inducible NOS) have been implicated in AIS, iNOS in particular contributes to neuroinflammatory processes (Iadecola et al., 1995). Inhibition/knockdown of the iNOS enzyme has been shown to be neuroprotective, and separate studies in which iNOS generation was indirectly reduced have supported this hypothesis (Zhao et al., 2000; Han et al., 2002; Coughlan et al., 2005; Park et al., 2006). Due to a possible role in both neuroprotection and neurotoxicity, a number of NO-based therapies have been suggested for the treatment of AIS, including NO donors, l-arginine (NO precursor), and iNOS inhibitors (Chen et al., 2017). To date, only glyceryl trinitrate (GTN), an NO donor commonly used in the management of angina, has progressed to clinical trials. A recent systematic review, however, highlighted that despite reductions in blood pressure, there is insufficient evidence to recommend GTN in AIS (Bath et al., 2017). Further pre-clinical evidence is required before other NO-based candidate therapies can progress to the clinical stage.

### Pro-Inflammatory Cytokines

The levels of IL-1β in the brain are upregulated over 40-fold within the first 24 h after AIS (Clausen et al., 2005). The pathogenic role of IL-1β in rodent models of stroke is well established; exogenous administration of IL-1β increased brain oedema, while IL-1α/β double-knockout mice showed ameliorated infarct size (Boutin et al., 2001). Separately, deficiency or inhibition of the IL-1 receptor (IL-1R1) improved long-term functional outcomes (Basu et al., 2005). Similarly, overexpression or treatment with IL-1Ra proved neuroprotective (Yang et al., 1997; Mulcahy et al., 2003). A recent systematic review concluded that anakinra, a recombinant IL-1 receptor antagonist (IL-1Ra), reduced infarct volume by 36.2% in experimental models of ischaemic stroke (McCann et al., 2016). In human studies, a phase II trial in 2005 showed anakinra to be safe and well tolerated in AIS patients (Emsley et al., 2005). With the replacement of the intravenous formulation by a subcutaneous injection, the effects of anakinra have since been re-investigated at a twice-daily dose (Sobowale et al., 2016). Although anakinra significantly decreased plasma levels of IL-6 and C-reactive protein, a possible negative interaction between IL-1Ra and tPA was revealed (Smith et al., 2018). Further pre-clinical and clinical studies are therefore required to determine whether anakinra or other IL-1 based therapies could be safely used as an adjunct in AIS treatment. Separately, reports of a potential neuroprotective role of IL-1β post-stroke must be re-examined (Amantea et al., 2010). Suggested mechanisms for this beneficial effect include the IL-1β-mediated release of high-mobility group box 1 (HMGB1) from reactive astrocytes as well as the production of neurotrophic factors (Hayakawa et al., 2010; Hewett et al., 2012).

TNF-α can be found in the CSF and serum of patients within 24 h of the onset of ischaemia (Zaremba and Losy, 2001; Lambertsen et al., 2012). The circulating levels of TNF-α are correlated with infarct size and the severity of neurological impairment. Inhibition of TNF-α also protects the brain against ischaemic injury (Chiba and Umegaki, 2013). Conversely, pre-treatment with recombinant TNF-α proved neuroprotective, while mice lacking TNF-α receptors showed larger infarct size (Bruce et al., 1996; Ginis et al., 2002). Arguably, the action of TNF-α depends on timing. In the early stages of stroke, TNF-α promotes BBB breakdown, leukocyte infiltration, and brain oedema, while further along the timeline, it mediates neuronal and microvasculature repair (Amantea et al., 2009). The location of TNF-α release could also be a factor; increased production in the striatum, for instance, promotes neurodegeneration, whereas release in the hippocampus promotes neuroprotection (Sriram and O’Callaghan, 2007). Separately, receptor subtype, gene polymorphism, or cell signalling pathways could determine what type of response TNF-α causes (Pradillo et al., 2005; Cui et al., 2012). To date, no clinical trials of TNF-α-based therapies in AIS have been published. However, etanercept, a biologic TNF-α inhibitor, has been studied experimentally in the setting of traumatic brain injury and clinically for the treatment of stroke-induced neurological dysfunction (Wang et al., 2013). In both studies, etanercept attenuated motor deficits. In humans, a single perispinal dose also improved psychological outcomes and reduced post-stroke complications (Tobinick et al., 2014). As TNF-α receptor inhibitors, such as etanercept, are already licensed for the treatment of certain autoimmune diseases, clinical trials in AIS could be expedited. The possibility of anti-TNF-α therapies leading to increased infection rates in the setting of stroke, however, would have to be addressed (Zeng et al., 2013).

### Cyclooxygenase/Lipoxygenase Pathways

Arachidonic acid metabolites from the COX or lipoxygenase (LO) pathways play a role in stroke injury (Yagami et al., 2016). Increased phospholipase A2 (PLA2) activity has been documented in several experimental models of stroke, while mice lacking PLA2 showed improved disease outcomes (Bonventre et al., 1997; Muralikrishna Adibhatla and Hatcher, 2006). Inhibition of COX-1/COX-2 enhanced survival of neurons in mouse hippocampus, suggesting the enzyme has a neurotoxic activity (Candelario-Jalil et al., 2003). Likewise, the delayed administration of COX-2 inhibitor NS398 reduced infarct volume and neurological deficits in an experimental model of stroke, whereas COX-2 overexpression exacerbated ischaemic injury (Doré et al., 2003; Sugimoto and Iadecola, 2003). Development of COX-2 inhibitors for the treatment of cerebral ischaemia was hindered by the finding that long-term use of COX-2 inhibitors themselves increased the risk of stroke (though strategies to prevent this adverse effect may yet be achieved) (Huang et al., 2016). Separately, leukotriene C4 (LTC4), an end-product of the 5-LO enzyme pathway, may play a more pronounced role in BBB dysfunction than do products of the COX pathway, with a biphasic expression pattern closely correlated to BBB opening (Rao et al., 1999). Use of a 5-LO inhibitor in an experimental model of transient ischaemia led to decreased brain levels of LTC4 and reduced cerebral oedema (Baskaya et al., 1996). Likewise, montelukast, a cysteinyl leukotriene receptor-1 antagonist licensed clinically for use in asthma, ameliorated hippocampal injury (Saad et al., 2015). The protective effect, however, could not be replicated in 5-LO knockout mice, casting doubt on leukotriene involvement in infarct development (Kitagawa et al., 2004). As for the COX pathway, a possible neuroprotective function has been argued. COX-1-deficient mice, for example, showed increased susceptibility to ischaemic injury, possibly as a result of compromised CBF (Iadecola et al., 2001). Several studies have highlighted a neuroprotective effect of prostaglandin E1, while accumulating evidence also demonstrates the beneficial effects of Prostaglandin E2 receptor 2/4 (EP2/EP4) receptor activation (Huang et al., 2016; Ling et al., 2016). The development of pharmacological agents targeting arachidonic acid (AA) metabolites in stroke may therefore yet move away from COX-2 inhibition towards manipulation of downstream EP receptors in the acute post-stroke phase.

### Matrix Metalloproteinases

MMPs, a family of zinc-binding endopeptidases, become dysregulated in the setting of AIS. Increased MMP activity leads to neuronal apoptosis, BBB breakdown, leukocyte infiltration, brain oedema, and, under certain circumstances, haemorrhage (Rosell and Lo, 2008). Various MMPs, including MMP-2, MMP-3, MMP-7, and MMP-9, have been implicated in infarct growth. MMP-2, which is constitutively expressed in the brain, contributes to ischaemic damage in the early stages (<24 h) through extracellular matrix disruption, tight junction protein degradation, and oxidative stress injury (Gasche et al., 2001; Yang et al., 2007). Conversely, MMP-9, an inducible enzyme secreted by microglia, macrophages, and infiltrating neutrophils, mediates the more intense and irreversible damage to the BBB associated with haemorrhage (Montaner et al., 2001). MMP-9, upregulated 15–48 h after the onset of ischaemia, has also been intimately linked to stroke pathology, with higher serum levels, a predictor of poor disease outcome (Abdelnaseer et al., 2017). Studies of MMP-9 knockout/inhibition highlighted decreased infarct volume and an overall reduction in ischaemic damage (Asahi et al., 2001; Gu et al., 2005). The same neuroprotective effect, however, was not noted in mice lacking MMP-2, leading to the theory that the enzyme is instead involved in post-stroke repair (Lucivero et al., 2007). Indeed, a wealth of evidence now points to a role for MMPs in processes such as neovascularization and neurovascular remodelling (Lee et al., 2006, Zhao et al., 2006). Treatment with MMP inhibitors at 7 days, for example, increased brain injury, impaired functional outcome, and reduced remodelling of the infarct site (Zhao et al., 2006). As a result, the clinical translation of therapies targeting MMPs may involve administration of modulators in the subacute stages of stroke that promote longer-term recovery. Separately, studies suggest MMP-9 is responsible for haemorrhagic transformation associated with tPA therapy, as levels of the enzyme are elevated in response to thrombolysis (Ning et al., 2006). As a result, MMP-9 inhibitors (e.g., minocycline) may yet find a role in the acute phase of AIS therapy as adjuncts to lengthen the therapeutic window for thrombolysis (Murata et al., 2008).

### Chemokines

Chemoattractant molecules are expressed by a range of cell types in AIS, including circulating immune cells, activated microglia, endothelial cells, astrocytes, and neurons (Kim et al., 1995b; Che et al., 2001). Chemokines, the expression of which are upregulated post-stroke, play a detrimental role in the early stages through leukocyte infiltration, ROS production, and BBB disruption (Kawabori and Yenari, 2015; Chen et al., 2018a). Mice lacking CCL5 or the neuronally expressed CX3CL1 (fractalkine), for example, showed reduced infarct size (Soriano et al., 2002; Terao et al., 2008). Similarly, inhibition of CXCL8 provided neuroprotection in transient brain ischaemia (Garau et al., 2005). Likewise, the genetic deletion of both CCR2 and CX3CR1 receptors proved neuroprotective in a ferric chloride model of ischaemia (Cisbani et al., 2018). However, evidence now also points to a second, neuroprotective function for chemokines in AIS, possibly as a result of increased stem cell recruitment to the infarct site (Wang et al., 2002). Several therapeutic strategies targeting chemokines—including small molecule receptor antagonists, neutralizing antibodies, pathogen-derived receptor antagonists, and modified chemokines—have been trialled in AIS, with moderate success (reviewed in Mirabelli-Badenier et al., 2011). Administration of an anti-MIP-3α antibody in rats, for example, reduced infarct size while TAK-779, a nonpeptide CCR5 antagonist, protected mice against focal cerebral ischaemia (Takami et al., 2002; Terao et al., 2009). Similarly, the pan-chemokine specific inhibitor NR58-3.14.3 also improved outcome in experimental stroke, while anti-CCL2 and CXCL8 antibodies decreased brain oedema and BBB permeability, respectively (Mirabelli-Badenier et al., 2011). Furthermore, the viral peptide, macrophage inflammatory protein-1α (MIP-II) (a chemokine analogue of MIP-II encoded by human herpesvirus-8 DNA), proved neuroprotective against focal cerebral ischaemia in mice (Takami et al., 2001). Despite these successes, however, issues preventing the clinical translation of chemokine-targeted therapies remain, including the correct timing of administration and drug concentration. While some research has shown the benefit of broad-spectrum chemokine inhibition, the risks that blanket-blocking the system poses to host defence and neuroprotective immune cell recruitment has also yet to be resolved (Lee et al., 2015). As a result, drugs such as JWH-133, which inhibit CXCL2-mediated neutrophil migration to the CNS without affecting the response in the periphery, may provide the way forward (Mirabelli-Badenier et al., 2011).

## Therapeutic Strategies Targeting Leukocyte Infiltration

E-, L-, and P-selectins, a family of transmembrane glycoproteins, have all been implicated in leukocyte trafficking in AIS (Yilmaz and Granger, 2008). In animal models, the selective blockade of E- or P-selectin resulted in improved neurological outcome (Huang et al., 2000; Mocco et al., 2002). Similarly, P-selectin-deficient mice showed decreased BBB breakdown (Jin et al., 2010). However, the use of an anti-L-selectin antibody in a rabbit model of stroke did not reduce ischaemic injury (Yenari et al., 2001).

Among the immunoglobulin superfamily, ICAM-1 and VCAM-1 have been the most studied in the setting of AIS (Stanimirovic et al., 1997). These molecules facilitate the adhesion of leukocytes to the endothelial wall and extravasation into the infarct site. The results of both *in vitro* experiments and human studies confirm increased ICAM-1 expression under conditions of ischaemia (Hess et al., 1994; Bitsch et al., 1998). As ICAM-1 levels peak at 12–48 h post-stroke, they are ideal targets for the acute phase. Studies of ICAM-1 deficiency/inhibition showed reduced ischaemic injury and decreased leukocyte infiltration (Kitagawa et al., 1998; Kanemoto et al., 2002; Vemuganti et al., 2004). ICAM-1 knockout mice also displayed reduced infarct size, as well as improvements in CBF and neurological function (Connolly et al., 1996). A phase III clinical trial of anti-ICAM-1 antibody enlimomab, however, failed to replicate these results, with the antibody-treated group reporting higher mortality (Investigators, 2001). Increased neutrophil activity in enlimomab-treated patients has been attributed to the antigenicity of the murine antibody used. As a result, the benefit of ICAM-1 modulation in AIS awaits confirmation. Likewise, there remains doubt as to the validity of VCAM-1 as an immunomodulatory target. Several groups have shown improved stroke outcomes in association with decreased VCAM-1 expression (Zhang and Wei, 2003; Cervera et al., 2004). A study of VCAM-1 knockdown, for example, led to decreased T lymphocyte infiltration and reduced infarct volume (Liesz et al., 2011). However, use of an anti-VCAM-1 antibody did not prove neuroprotective in either rats or mice, casting further doubt on the viability of this target in AIS (Justicia et al., 2006).

Integrin molecules, such as leukocyte function-associated antigen-1 (LFA-1 or CD11a/CD18), macrophage-1 (Mac-1 or CD11b/CD18), and very late adhesion molecule-4 (VLA-4 or CD49d), facilitate the binding of leukocytes to areas of activated endothelium in AIS (Iadecola and Anrather, 2011). The expression of CD11a/CD18 is upregulated in stroke patients (Kim et al., 1995a). In an experimental model of CD11a/CD18 deficiency, reductions in infarct volume and neurological deficit were noted (Arumugam et al., 2004). Administration of an anti-CD11b antibody produced the same result (Chen et al., 1994). Likewise, in mice lacking CD18 or Mac-1, reduced infarct size, decreased neutrophil infiltration, and decreased mortality were shown (Prestigiacomo et al., 1999; Soriano et al., 1999). The use of Hu23F2G, an anti-Mac-1 antibody, caused a similar amelioration of ischaemic injury (Yenari et al., 1998). In the phase III clinical trial “LeukArrest,” however, Hu23F2G did not prove neuroprotective (Becker, 2002). A second human study—Acute Stroke Therapy by Inhibition of Neutrophils (ASTIN)—instead investigated the effect of Mac-1 inhibitor, UK-279,276 (neutrophil inhibitor factor), in AIS patients (Krams et al., 2003). Again, however, despite good pre-clinical evidence, ASTIN failed to meet the predetermined endpoint of a 3-point additional mean recovery on the Scandinavian Stroke Scale, and the study was terminated early (Jiang et al., 1995). Plausible confounding factors in both trials include the timing of drug administration, differences in integrin molecules between humans and rodents, and the variability in reperfusion in human stroke vs. animal models (Jickling et al., 2015). On this last point, it has been noted that anti-integrin therapies provide more benefit in transient compared with permanent models of ischaemia in animals (Zhang et al., 1995). The observation that an anti-Mac-1 antibody extends the therapeutic window of tPA suggests a possible place for these therapies as an adjunct in AIS treatment (Zhang et al., 2003). Another integrin-based therapy, which has progressed to the clinical trial stage is natalizumab—an anti-VLA-4 antibody used to attenuate neuroinflammation in relapsing-remitting MS. The “ACTION” trial (phase II) explored the safety and efficacy of natalizumab in stroke (Elkins et al., 2017). While natalizumab did not affect infarct volume, improvements in functional outcomes at 30 days warranted further investigation. The follow-up phase IIb trial, however, did not meet its primary or secondary endpoints. This likely concludes the investigation into natalizumab in AIS at this time.

Perhaps, as a result of poor clinical translation, research into strategies targeting leukocyte infiltration in AIS has shifted away from global inhibition to specific subsets. Therapies aimed at reducing T-cell extravasation, for example, have received increased interest. Fingolimod, a sphingosine-1-phosphate receptor modulator used in the treatment of relapsing-remitting MS, is one such treatment. A wealth of pre-clinical evidence now supports the benefits of fingolimod on AIS outcome (Liu et al., 2013). The suppression of T-cell infiltration into the CNS plays a role in this efficacy, though other mechanisms such as decreased BBB dysfunction, reduced microglial activation, and direct neuroprotection are also likely to be involved (Kraft et al., 2013; Li et al., 2016). At the clinical level, fingolimod has been investigated in a small number of pilot studies (either alone or in combination with thrombolysis). Both studies confirmed a beneficial effect of fingolimod on infarct size, rates of haemorrhagic transformation, and neurological function (Fu et al., 2014b; Zhu et al., 2015).

## Stroke-Induced Immunodepression

The early activation of the peripheral immune system post-stroke gives way 2 days after initial ischaemia to a second phenomenon termed stroke-induced immunodepression (SIID). Theoretically, SIID may have evolved as an adaptive response, protecting the CNS from harmful autoimmune responses. The active depression of immune defence mechanisms, however, dramatically increases the susceptibility of the host to infection. Indeed, large studies have estimated approximately 30% of stroke patients contract infections, the most prevalent of which are urinary tract infections (UTIs) and pneumonia (Westendorp et al., 2011). Microorganisms routinely implicated in post-stroke infection include *Staphylococcus aureus*, *Pseudomonas aeruginosa*, *Klebsiella pneumoniae*, and *Escherichia coli*. Anatomical factors seem to be important in post-stroke infection. The extent of lesion size, for example, is associated with occurrence of pneumonia, though whether infarct location influences events remains unclear (Minnerup et al., 2010). The fact that most cases of pneumonia and UTI are diagnosed within the first few days after stroke, however, implies SIID is involved and that infections are not simply a manifestation of poor patient care (Westendorp et al., 2011). Regarding outcome, almost all major studies of post-stroke infection have shown a significant impact of infection on functional outcome and patient mortality (Vermeij et al., 2018). As a result, strategies that aim to prevent or cure such infections are currently receiving greater interest.

SIID is typified by lymphopenia, increased levels of anti-inflammatory cytokines such as IL-10 and transforming growth factor beta 1 (TGF-β), and splenic atrophy (Kamel and Iadecola, 2012). Mechanistically, the CNS causes immunological changes through a variety of humoral and neural pathways, including the sympathetic nervous system (SNS), the parasympathetic nervous system (PNS), and the hypothalamic–pituitary–adrenal (HPA) axis (Brambilla et al., 2013). Glucocorticoids, end-products of HPA axis activation, curb the peripheral immune response post-stroke through promoting lymphopenia, impairing lymphocyte/neutrophil function, and deactivating macrophages (Prass et al., 2003; Mracsko et al., 2014). Glucocorticoid receptor blockade reduced splenocyte apoptosis and corrected lymphopenia in an experimental model of stroke, though this antagonism did not prevent pneumonia (Prass et al., 2003). The beneficial effect of glucocorticoid blockade on lymphocyte counts was confirmed in a further mouse study, though this intervention did increase levels of the SNS mediator, metanephrine (Mracsko et al., 2014). Both of these studies suggest that modulation of the glucocorticoid pathway can reduce the manifestation of SIID, though whether this leads to reduced incidence of infection or deleterious signalling elsewhere still needs to be reconciled.

Increased transmission in the PNS, chiefly through vagal nerve activity, is also capable of altering peripheral immune function. Studies have shown the detrimental effect that acetylcholine (ACh), secreted by both parasympathetic nerves and splenic memory T cells, has on macrophage function (Rosas-Ballina et al., 2011; Trakhtenberg and Goldberg, 2011). Activation of nicotinic acetylcholine receptor α7 (nAChRα7) results in decreased pro-inflammatory cytokine secretion, predisposing the host to infection (Rosas-Ballina et al., 2011; Trakhtenberg and Goldberg, 2011). As of yet, however, therapies targeting the PNS have focused more on reducing innate immune function at the infarct site (Kox and Pickkers, 2015).

One branch of the autonomic nervous system that modulates peripheral immune function post-stroke and has progressed to human studies is the SNS. Increased levels of adrenaline and noradrenaline post-ischaemia skews leukocytes towards an anti-inflammatory phenotype, while also disabling hepatic invariant natural killer T cells (iNKT) in mice (Chamorro et al., 2012). The latter cell type in particular has been shown to be pivotal in host defence against post-stroke infection (Wong et al., 2011). The inhibition of iNKT function, however, can be reversed pharmacologically through administration of the glycolipid, α-galactosylceramide, or beta blockers such as propranolol (Shi et al., 2018). In the clinic, the beneficial effects of beta blockers on infection rates and early mortality have been replicated several times (Dziedzic et al., 2007; Sykora et al., 2015). Controversially, separate studies showed a negative effect of beta blockers, coupled with higher rates of adverse events (Westendorp et al., 2016). In theory, beta blockers could affect CBF or aggravate pro-inflammatory responses in the brain. Evidently, further studies into immunomodulatory therapies of the SNS are required.

In terms of post-stroke infection outcomes, the most recent systematic reviews of prophylactic antibiotic therapy suggest that this approach is not effective (Vermeij et al., 2018). Despite a meta-analysis finding that preventative antibacterial drugs reduced overall infection rates, no effect on functional outcome (modified Rankin scale) or mortality was seen (Zheng et al., 2017). Importantly, however, the pathophysiology of post-stroke infection is still poorly understood, while certain classes of antibiotics (e.g., fluoroquinolones) have known neurotoxic properties. The use of prophylactic antibiotic therapy, therefore, may yet prove effective in certain patient subgroups. Nevertheless, in order to achieve improved functional outcomes, a combination of antibiotics and immunomodulatory drugs could show greater promise.

## Brain Antigen Tolerization

Increased permeability in the BBB, coupled with significant levels of cell death, results in greater exposure and presentation of brain antigens to the immune system. This can lead to the generation of autoimmune B- and T-cell responses. A likely function of SIID is to reduce the number of auto-reactive IFN-γ-secreting T-cells; this was demonstrated in a 2D2 transgenic mouse model where the T-cell receptor is specific for myelin oligodendrocyte glycoprotein (MOG), a CNS antigen (Römer et al., 2015). Clinical evidence demonstrates a strong correlation between Th1 responses and poorer stroke outcome up to 3 months later in humans (Becker et al., 2011). The definition of Th1 responses in this clinical study was a higher ratio of IFN-γ to TGF-β secretion by T-cells when stimulated with a range of self-antigens including myelin basic protein (MBP); however, the magnitude of each of these responses was not reported. Modulation of immunity towards a Th1 phenotype is favoured during the inflammatory response to bacteria; Th1 responses to MBP, in particular, were more prevalent in stroke patients with pneumonia (Becker et al., 2011). It is unclear if these autoreactive cells are involved in short- or long-term pathogenesis or, instead, act as a severity marker or are associated effects of the stroke. Some studies suggest a role of autoreactive Th1 in poor outcome (Zierath et al., 2010), whereas other studies demonstrate no impact (Römer et al., 2015). However, the self-antigen specificity of the response is clear as memory immunity to foreign antigens such as tetanus toxoid did not associate with outcome (Becker et al., 2011). It is possible that a Th1 response could indeed be driving T-cell cytotoxicity in the brain, similar to other autoimmune pathologies, such as type 1 diabetes. Immune-based therapies that modulate the T-cell phenotype away from a cytotoxic response towards a more tolerized response, possibly mediated by Treg, might have application in stroke.

Previous therapies attempted to induce antigen-specific tolerance to E-selectin or to MBP *via* repeated mucosal administration. Induction of a Treg response to MBP in Lewis rats before stroke resulted in an improved outcome at early, but not late (3 months) time points after stroke (Gee et al., 2009). This could be due to the inherent plasticity of T-cell phenotypes, particularly between Treg and Th17, as the latter type is known to be pathogenic in autoimmune CNS diseases [such as the MS mouse model and experimental autoimmune encephalomyelitis (EAE)] (Jadidi-Niaragh and Mirshafiey, 2011). Increased IL-6 expression during infection could promote Treg cells to switch to a more Th17 function (Kimura and Kishimoto 2010). E-selectin is only expressed in blood vessels upon activation. It has therefore been hypothesized that induction of a regulatory T-cell response to E-selectin would be specifically directed to blood vessels undergoing activation in the brain and thereby reduce the risk of stroke (Hallenbeck, 2010). However, induction of mucosal tolerance requires repeated low-dose administration and takes time to develop. Although pre-clinical results provided some proof-of-principle and clinical studies were initiated in 2003 and terminated in 2010 (NCT00069069), no clinical data on safety or potency have yet been published. Overall, induction of antigen-specific Treg using mucosal tolerance has suffered from logistical (repeated doses required) as well as clinical efficacy issues.

Non-antigen-specific approaches are also being evaluated. These cell therapies could act in a more systemic, regenerative manner by targeting reparative, anti-inflammatory processes, which contribute to recovery from disease and co-morbidities and thereby may have a multi-dimensional effect on disease. Phase II/III clinical trials are ongoing in ischaemic stroke patients to assess MultiStem, *in vitro* expanded multipotent adult progenitor bone marrow cells that do not require human leukocyte antigen (HLA) matching (Hess et al., 2014; Osanai et al., 2018). Favourable safety and tolerability have been observed (Hess et al., 2014), with a time window (24–36 h) beyond that of recombinant tissue plasminogen activator (rt-PA) and endovascular thrombectomy. MultiStem treatment did not demonstrate a significant difference to placebo treatment for the primary endpoint of Global Stroke Recovery Assessment or secondary endpoints relating to disability, neurologic deficit, and activities of daily living. However, some (15.4%) patients achieved an improved outcome, and life-threatening adverse events and mortality were lower in the treated group. MultiStem-treated patients showed a significant decrease in the number of circulating CD3+ T-cells, possibly suggesting a decrease in inflammation. Earlier treatment (24–36 h post-stroke) exhibited more favourable outcome than did delayed treatment. Therefore, although the trial failed to achieve the primary or secondary outcomes, it provides positive evidence of the potential of cell-therapy-based approaches, including multipotent adult progenitor cells (MAPCs), while also suggesting that timing is important to success (Mays and Deans, 2016). Elsewhere, various cell immunotherapy strategies are being assessed to treat other autoimmune diseases. Adoptive transfer of *ex vivo* expanded polyclonal Treg for type 1 diabetes, for example, has been demonstrated to be safe, underlying future clinical testing (Bluestone et al., 2015).

Alternatives to adoptive cell transfers are also being developed. Modulating the IL-2/CD25 axis in favour of Treg growth and away from activated and/or effector T-cells has been shown to be safe and potent in clinical trials across 11 autoimmune diseases (Rosenzwajg et al., 2018). It is now being clinically assessed in patients with stable ischaemic heart disease and acute coronary syndromes (NCT03113773) (Zhao et al., 2018). Repeated systemic administration of tolerizing-inducing formulations, such as nanoparticle-based delivery of aryl hydrocarbon receptor ligands and β-cell antigens, has demonstrated pre-clinical efficacy in a mouse model of type 1 diabetes (Yeste et al., 2016). Given that the pathology of ischaemic stroke has clear differences with these more chronic autoimmune diseases, with respect to the involvement of rapid immune depression and infection, successful immunotherapies for other autoimmune disease may not directly translate to AIS. However, lessons learned from all of these cell and immune therapies should be beneficial in developing efficacious immunotherapies that re-establish tolerance for long-term protection against stroke and potentially prevent decreased cognition in the stroke patient.

## Regulatory Immune Cell Strategies

Peripheral immune cells, which may contribute to the repair of the injured brain, include cells of both the innate and adaptive immune systems. M2- but not M1-type macrophages have been considered as promising target to enhance angiogenesis and neurovascular unit remodelling after stroke by producing factors such as vascular endothelial growth factor (VEGF), IL-8, Insulin-like growth factor 1 (IGF-1), and TGF-β (Willenborg et al., 2012; Nakamura et al., 2016). Neutrophils, although typically considered cells that induce damage in the acute phase of stroke (Amulic and Hayes, 2011; Herz et al., 2015), can also be involved in neural network remodelling in the latter stages. Through degradation of extracellular matrix, neutrophils release VEGF and TGF-β. In addition, neutrophils possess the ability to clear dead cells, debris, and bacteria, creating a better microenvironment for repair (Zlokovic, 2006; McDonald et al., 2010; Corps et al., 2015). With the use of genetically modified animals or lymphocyte depletion in animal stroke models (Rag^−/−^ and SCID mice), the important role that lymphocytes play has been shown. Animals lacking lymphocytes had significantly smaller infarcts than had wild-type (WT) mice (Yilmaz et al., 2006; Hurn et al., 2007). Depletion of T-cells (both CD4+ and CD8+ subtypes) significantly reduced infarct volumes. Depletion of B-cells, on the other hand, had no effect on the infarct volumes 24 h after stroke induction (Yilmaz et al., 2006). This could indicate that T-cells but not B-cells play a role in post-stroke neural damage. Interestingly, in a separate study, B-cell knockout mice (µMT^−/−^ mice) showed larger infarcts than did WT mice, suggesting that B-cells may have a protective function (Ren et al., 2011). Specifically, one subset of B-cells, IL-10-producing regulatory B-cells, may play an important role in neuroprotection.

Regulatory B-cells were shown to play a protective role in autoimmune animal models such as MS (EAE), rheumatoid arthritis [collagen-induced arthritis (CIA)], type 1 diabetes, and systemic lupus erythematous by dampening pro-inflammatory T-cells and enhancing the expansion of regulatory T-cells (Yang et al., 2013; Tedder, 2015). The main protective effect of regulatory B-cells is mediated through IL-10 production. The role of regulatory B-cells in stroke is still under investigation; however, a study where transfer of WT B-cells into B-cell knockout mice, when compared with mice receiving IL-10^−/−^ B-cells, 24 h before the induction of stroke showing reduced infarct volume suggests a strong role of IL-10-producing B-cells (Ren et al., 2011). Chen et al. have shown that CD5+CD1dhi B10 cells are enriched in the ipsilateral versus contralateral hemisphere of mice at 48 h post-stroke, as well as circulating regulatory B-cells (Chen et al., 2012). Adoptive transfer of *in vitro*-induced regulatory B-cells to mice 24 h before the induction of stroke resulted in increased regulatory T-cell subsets (CD4+Foxp3+ and CD8+CD122+). Compared with that of animals without stimulated B-cells, infarct volume decreased (Bodhankar et al., 2015). If regulatory B-cells were to be considered for adoptive transfer in stroke, however, the purity of the population would need to be very high. Otherwise, non-regulatory B-cells could exacerbate post-stroke damage through antibody-mediated neurotoxicity (Doyle et al., 2015).

Regulatory T-cells are an important cell subset playing an active regulatory role in the brain after stroke. These cells (CD25+Foxp3+), which constitute approximately 10% of peripheral CD4+ T-cells, are essential for the promotion of immunosuppression through production of cytokines such as IL-10, TGF-β, and IL-35 (Zouggari et al., 2009; Wan, 2010). The protective role of Tregs after cerebral ischaemic injury has been shown in multiple studies (Liesz et al., 2009; Li et al., 2013; Liesz and Kleinschnitz, 2016). Regulatory T-cells function through dampening excessive immune responses and also through promoting the generation of new neuroblasts (Wan, 2010). Induction of immunosuppressive environments by recruitment of suppressive immune cells after ischaemia can reduce inflammation and decrease neural injury. The challenge of the low frequency of regulatory cells, however, has thus far restricted their clinical utility. In addition, questions remain as to how and when Tregs move from the periphery to the brain, a factor that may determine the therapeutic window. Although *in vivo* studies in mice have shown that it is possible to increase the frequency of regulatory cells [e.g., treatment with IL-2/IL-2 antibody complex increased Treg and protected against transient ischaemic attack (TIA)], the safety of this therapy in patients has yet to be established (Zhang et al., 2018). Further studies are needed to fully understand both the timing and frequency of suppressive cell infiltration into the infarcted brain. The methods of regulatory cell induction will also be crucial to evaluate in a clinical setting. Overall, a more comprehensive understanding of regulatory immune responses at different stages of stroke pathology will aid the design of safer and more effective immunomodulatory strategies.

## The Role of the Gut in Stroke: Targeting Peripheral Immune Systems

The bidirectional brain–gut interaction has received increased attention with respect to several neurologic and psychiatric diseases. Gut microbiota signal to the CNS *via* neural, immunologic, hormonal, and metabolic pathways, whereas the CNS alters the intestine by regulating gut motility and secretion as well as *via* the enteric nervous system. Maintenance of this symbiosis is critical to health; for example, unwanted gut permeability and influx of bacterial components and other danger-signalling molecules (DAMPs) can activate the immune system. At worst, such infiltration can cause bacteraemia, sepsis, and death.

An association between brain autoimmunity and intestinal microbiota was first discussed with respect to EAE (Wekerle, 2017). Pre-clinical studies that assess the impact of the gut microbiota on CNS damage generally involve the use of germ-free (GF) mice or treatment with antibiotic cocktails to deplete drug-sensitive bacterial strains. With respect to stroke, it is proposed that infarcted brain tissue can affect intestinal function and immunity *via* the adrenergic system and/or HPA axis. It has been reported that both T- and B-cell numbers are significantly reduced in Peyer’s patches in mice post-stroke, suggesting a link between the CNS and the gut-associated lymphoid tissue (GALT) (Schulte-Herbrüggen et al., 2009). Increased intestinal permeability may promote bacterial invasion to non-gut organs. Increased bacterial load in mesenteric lymph nodes has been reported after MCAO (Caso et al., 2009). In a mouse model of ischaemic stroke, post-stroke infection was not seen in mice born and raised in GF facilities but was observed in those housed from birth in specific-pathogen-free (SPF) facilities. It was demonstrated in the same study that the source of disseminated bacteria in the infected cases was indeed the host small intestine (Stanley et al., 2016). Such bacterial translocation also induces inflammation and promotes Th1 and Th17 responses, all of which are normally required to clear a systemic bacterial infection. As discussed previously, however, these Th1 responses are associated with poorer stroke outcome. An elegant paper by Singh *et al.* showed how large stroke lesions lead to gut microbiota dysbiosis, which in turn worsens stroke outcome *via* immune-mediated mechanisms including intestinal lymphocyte migration to the ischaemic brain (Singh et al., 2016). Faecal microbial transplantation, however, normalizes this stroke-induced dysbiosis and improves stroke outcome. In the same study, it was highlighted that stroke specifically reduces microbiota species diversity and causes overgrowth of Bacteroidetes, a phenomenon associated with intestinal barrier dysfunction and reduced intestinal motility. Effectively, the microbiota composition may impact stroke severity. For example, altering the ratio of Firmicutes to *Bacteroides* in young mice to resemble the ratio observed in old mice before MCAO increased mortality and decreased behavioural performance (Spychala et al., 2018). An “aged” microbiota composition enhanced systemic inflammation when transplanted into young mice. Transplanted mice also had lower levels of short-chain fatty acids (SCFAs). These bacterially produced small molecules are important modulators that stabilize the gut epithelial barrier and modulate immune responses (Spychala et al., 2018). This study again suggests that the gut microbiota can affect stroke outcome in a mouse model. Aberrations to gut microbiota may not only significantly decrease survival, however, but also lead to acute colitis and other post-stroke sequelae (Winek et al., 2016a). However, translational caveats inherent to microbiota research in general are also true for stroke research. The differences in the anatomy, physiology, genetic heterogeneity, diet, and housing of mice compared with humans must be appreciated to prevent incorrect translation of findings in rodent gut microbiota models to human disease (Winek et al., 2016b). Instances where antibiotic-induced alterations to the gut microbiota reduced ischaemic injury should also be further explored (Benakis et al., 2016). Overall, more clinical studies are required to truly evaluate the role of the microbiota in post-stroke responses and to define strategies of how this response can be modified for improved patient outcomes.

## Ischaemic Preconditioning

Significant interactions between the stroke and the periphery are also at play in remote ischaemic preconditioning (RIPC). Originally discovered by Murray and colleagues, preconditioning is a procedure by which a noxious stimulus is applied to a tissue or organ below the threshold of damage (Murry et al., 1986). After a recovery period, organs such as the brain develop a tolerance to the same or even different noxious stimuli given above the threshold of damage. Various types of preconditioning stimuli have been used experimentally to protect the brain, heart, liver, kidney, and other organs. Chief among these are ischaemic preconditioning (in which the blood supply to a target organ is temporarily interrupted before introducing a longer infarct) and hypoxic preconditioning (in which animals are exposed to oxygen levels around 8% to 9% for a few hours). However, achieving ischaemic or hypoxic preconditioning in the brain itself would be more challenging and less practical than in other organs. Therefore, experimental and clinical evidence that shows robust tolerance can be achieved in the brain by inducing ischaemia–reperfusion in a distant (remote) non-vital organ has attracted a great deal of attention (Hess et al., 2013; Chen et al., 2018c). Both local preconditioning and RIPC protect the brain either rapidly after stimulation (known as early, first-window, or classical preconditioning) or after a 24-h delay to induce protection lasting at least 48 h (known as delayed or second-window preconditioning). The complex mechanisms underlying early and delayed protection differ, with evidence for both humoral and neurogenic mechanisms (many of which are shared between local preconditioning and RIPC). Most mechanisms (i.e., intracellular signalling cascades, role of mitochondria, and activation of neural pathways) are beyond the scope of this review, and the next section will focus instead on the evidence that inflammation and immunomodulation also play a role.

RIPC prevents BBB breakdown following stroke (Wei et al., 2012) and regulates inflammatory gene expression in the blood (Konstantinov et al., 2004), whereas blocking pro-inflammatory signalling pathways abolishes ischaemic tolerance to focal stroke in response to hypoxic preconditioning (Stowe et al., 2012; Selvaraj et al., 2017). Molecular inflammatory mechanisms underlying different brain preconditioning modalities were recently reviewed (Garcia-Bonilla et al., 2014), and there is a large body of evidence implicating the innate immune system in preconditioning (Amantea et al., 2015). The adaptive immune response may also play a role, as RIPC results in dramatic changes in T- and B-lymphocyte subsets in the spleen, while splenectomy attenuates the protective effect of RIPC on ischaemic brain injury (Chen et al., 2018b). Because similar neuroinflammatory pathways and lymphocytes underlie the pathophysiology of both MS, a progressive condition, and ischaemic stroke, which occurs more acutely (Paterno and Chillon, 2018), it is interesting to note that local preconditioning and RIPC also seem to be effective in the context of EAE (Camara-Lemarroy et al., 2018). Mice that underwent hypoxic preconditioning show fewer CD4+ T-cells and a delayed Th17-specific cytokine response, as well as increased numbers of Tregs and IL-10 following EAE induction (Esen et al., 2016). This effect on Tregs is reminiscent of observations in the kidney, where local ischaemic preconditioning leads to an increase in CD4+CD25+FoxP3+ and CD4+CD25+IL-10+ Tregs (Cho et al., 2010; Kinsey et al., 2010). These Treg cells seem to be able to suppress conventional T-cells with diverse antigen specificities (Corthay, 2009), suggesting that an increase in Treg number due to preconditioning in one organ might be protective in another and might therefore also play a role in RIPC. Alternatively, preconditioning might affect neutrophils (Shimizu et al., 2010), monocytes/macrophages (McDonough and Weinstein, 2016), or lymphocytes other than Tregs, in particular immunosuppressive B-cells (Monson et al., 2014). RIPC has been shown to alter the CD8+ T-cell, B-cell, NKT-cell, and monocyte response after stroke (Liu et al., 2016). Exercise-induced neuroprotection is another form of preconditioning that also seems to involve immunomodulation (Ding et al., 2005), and multiple reports point to a role of B-cells (reviewed in Selvaraj et al., 2016).

Because of the unpredictable nature of stroke, the clinical relevance of preconditioning is unclear, and RIPC is most likely to be performed under specific circumstances, such as during coronary bypass surgery or in patients undergoing carotid endarterectomy (Walsh et al., 2010). However, the potential clinical effectiveness of hypoxia- and ischaemia-induced tolerance to stroke is supported by the protective effect of living at higher altitude on stroke mortality (Faeh et al., 2009), and by observations that transient ischaemic attacks (TIAs) are associated with reduced severity of subsequent stroke (Weih et al., 1999). Furthermore, remote ischaemic conditioning performed during (“perconditioning”) (Schmidt et al., 2007) or after (“postconditioning”) (Kerendi et al., 2005) the ischaemic episode in the target organ also seems to be effective in animal models of myocardial infarction, suggesting that mechanisms underlying preconditioning might also be utilized after a stroke has occurred. Indeed, a recent study showing that remote ischaemic postconditioning administered 5 days after stroke reverses peripheral post-stroke immunosuppression and leads to a gradual functional improvement over an observation period of 3 months suggests that this approach not only may be protective but also may in fact promote recovery, lessening the importance of identifying the critical therapeutic time window (Doeppner et al., 2018).

## Conclusion

Despite the benefits that thrombolysis and mechanical thrombectomy offer in AIS care, new agents are now required both as adjuncts and as separate therapies. **Table 1** highlights various immunomodulatory therapeutic strategies considered in the treatment of AIS to date. In an era of increasingly complex biomedical research, such strategies feature not only small molecule drugs but also monoclonal antibodies and, more recently, cell-based therapies. Although advanced biological therapies offer several advantages over their chemical counterparts (e.g., increased target specificity and reduced toxicity), the issues of cost, patient access, formulation, and unexpected immune reactions still need to be reconciled in the setting of stroke.

A separate dichotomy identifiable in the list of immunomodulatory therapeutic strategies available is whether the mechanism underlying each treatment reduces neurotoxicity or instead promotes neurorestoration and tissue repair. Many of the drugs trialled to date (anakinra and etanercept) target innate immunity, a portion of the immune response responsible for widespread neurotoxicity in the acute phase of the disease. Arguably, however, even if such therapies eventually proved effective, their widespread clinical use could be limited to tPA adjuncts by time alone. Conversely, therapies affecting the adaptive immune response (Tregs and Bregs), involved predominantly in repair processes, could be administered over a longer therapeutic window. In this regard, therapeutic strategies not bound by time constraints (e.g., postconditioning) could offer the most flexibility of all.

One aspect of ischaemic preconditioning, which resonates strongly in current stroke research, is the effect that changes to the periphery could have on both the CNS and the post-stroke organism at large. In this regard, the development of drugs that could abrogate SIID or bolster the host defence against post-stroke infection has received intense focus, especially in the era of antibiotic resistance. Separately, the role of the gut microbiota and the brain–gut axis plays in stroke recovery has been elucidated. Here, novel therapies that could either control post-stroke dysbiosis or modify gut-induced neuroinflammation could improve stroke outcome. The skewing of the peripheral immune response with a view to reduced brain injury could also be exploited through the phenomenon of brain antigen tolerization.

As part of the last several decades of pre-clinical and clinical stroke research, a wealth of information on the immune involvement in AIS has been discovered. As a result, the field has progressed from the initial belief that some innate or adaptive immune components exacerbate ischaemic insult to the current understanding that many immune elements play multi-faceted roles in both stroke-induced damage and post-stroke repair. A greater appreciation of the intimate, bi-directional communication between the CNS and the immune system post-stroke has helped clarify how such functional polarization occurs. Meanwhile, the concept of SIID, potentially responsible for a significant portion of stroke patient mortality, has attracted attention to immunomodulatory therapeutic strategies in AIS. Given the shortcomings of neuroprotection trials, such strategies will no doubt continue to receive increased interest in the future.

## Author Contributions

KM, SA, AM, and CW wrote the original manuscript. All authors subsequently reviewed, edited, and agreed upon the final draft.

## Funding

This work was generously supported by a Government of Ireland Postgraduate Scholarship from the Irish Research Council (grant number GOIPG/2017/431), an HRB Health Research Award (HRA-POR-2015-1236), and a Marie Curie Career Integration Grant (631246).

## Conflict of Interest Statement

The authors declare that the research was conducted in the absence of any commercial or financial relationships that could be construed as a potential conflict of interest.
